# Composition of non-cyanobacterial diazotrophs in suspended and sinking particles is driven by productivity phase

**DOI:** 10.1128/spectrum.01386-26

**Published:** 2026-08-06

**Authors:** Christian Furbo Reeder, Lisa W. von Friesen, Josephin Lemke, Kristian Spilling, Hanna Farnelid

**Affiliations:** 1 Department of Biology and Environmental Science, Centre for Ecology and Evolution in Microbial Model Systems (EEMiS), Linneaus University 620860, Kalmar, Sweden; 2 Marine and Freshwater Solutions unit, Finnish Environment Institute (Syke) 73144, Helsinki, Finland; 3 Tvärminne Zoological Station, University of Helsinki, Hanko, Finland; 4 Centre for Coastal Research, University of Agder 4343, Kristiansand, Norway; Ocean University of China, Qingdao, Shandong, China

**Keywords:** particles, diazotrophs, Baltic Sea, productivity, nitrogen fixation, marine snow

## Abstract

Biological nitrogen fixation is a key process impacting productivity in the Baltic Sea. Most studies of this process have focused on filamentous cyanobacterial diazotrophs, leaving the ecological roles of non-cyanobacterial diazotrophs (NCDs) poorly resolved. Here, we investigate the diazotrophic community across different primary productivity phases (productive and post-productive) and within distinct particle fractions (suspended and sinking) across the Northern Baltic Proper, Gulf of Finland, and Gulf of Bothnia. Using *nifH* gene amplicon sequencing, we show that NCDs dominate the diazotrophic community, accounting for 81%–100% of all sequences, whereas filamentous cyanobacteria contribute up to 19% and are largely restricted to the post-productive phase. Productivity phases emerged as a descriptor for structuring the diazotroph community composition, with Thermodesulfobacteriota dominating during the productive phase (up to 64.8%) and Gammaproteobacteria during the post-productive phase (up to 77.1%). Suspended and sinking particles were broadly similar, yet each exhibited distinct taxon-specific enrichments between phases, which might suggest different ecological strategies within the major NCD groups. This suggests potential roles in aggregate-associated nitrogen fixation; such mechanisms could include polysaccharide degradation and anaerobic metabolism. Phytoplankton composition (18S rRNA) varied between productivity phases and co-occurred with shifts in the diazotrophic community, suggesting potential cross-domain interactions. Comparing across basins, we did not observe significant differences in diazotroph community composition, except for a higher relative abundance of cyanobacterial diazotrophs in the Gulf of Finland. Taken together, our findings show diverse NCD lineages inhabiting particle-associated niches, highlighting the need to further investigate their ecological significance in the Baltic Sea.

**IMPORTANCE:**

Nitrogen is a key nutrient that supports life in marine ecosystems. Traditionally, nitrogen fixation in the Baltic Sea has been attributed mainly to filamentous cyanobacteria. In this study, we show that non-cyanobacterial diazotrophs dominate the nitrogen-fixing community across productivity phases. We also demonstrate that suspended and sinking particles provide habitats for these microorganisms, allowing different groups to thrive under varying conditions. We further show that shifts in the phytoplankton community co-occur with changes in the diazotrophic composition. Together, our findings reveal that nitrogen fixation in the Baltic Sea extends beyond filamentous cyanobacteria and involves a diverse particle-associated diazotrophic community. Understanding the role of these overlooked microorganisms is important for predicting how nitrogen cycling will respond to ongoing changes, such as warming, eutrophication, and shifts in plankton communities.

## INTRODUCTION

Dinitrogen (N_2_) fixation is the major pathway to introduce bioavailable nitrogen (N) into the ocean. In the Baltic Sea, filamentous cyanobacteria are considered the main N_2_-fixers (1) and can sustain up to 90% of the net summer production, contributing 180–430 Gg N yr^−1^ (2). While the dominant role of cyanobacteria has been well documented, some studies have provided insights into the presence and potential significance of non-cyanobacterial diazotrophs (NCDs) (3–7). N_2_ fixation is a highly energetic process, and a shared feature among diazotrophs is the irreversible inhibition of nitrogenase in the presence of oxygen. Cyanobacterial energy demand is met through photosynthesis; however, NCDs do not have photosynthetic capabilities and depend on organic carbon for energy (8, 9), making them highly genetically and metabolically diverse, with multiple energy generation and nutrient transport mechanisms described (9).

Marine snow is aggregates of organic material derived from, e.g., phytoplankton and cellular debris (10). They play a pivotal role in carbon export and serve as a hotspot for biological activity (11). Marine particles (i.e., marine snow) are considered an important niche for NCD N_2_ fixation, due to their anoxic core and their relatively high content of organic and inorganic material, supplying labile carbon and nutrients for heterotrophic bacteria (8, 11–15). NCDs on particles have recently been reported from a wide range of marine environments, including different particle types in the North Atlantic (16), the North Pacific Ocean (17–19), and the upwelling regions in the eastern tropical South Pacific Ocean (20). NCDs are frequently enriched in large filter size fractions (21, 22). NCDs are known to actively colonize particles (23, 24), and recent studies have reported NCDs to colonize different particle fractions (suspended and sinking particles) separated by gravitation with marine snow catchers (16, 18), and to actively fix N_2_ on particles in the North Pacific (17, 18). In contrast, cyanobacterial diazotrophs were more common in the suspended particle fraction (16, 18). To date, there is no study investigating the presence of NCDs in different particle fractions in the Baltic Sea.

The Baltic Sea is a semi-enclosed, brackish water body characterized by strong north-south gradients in salinity, nutrient availability, and productivity. It has several sub-basins with different physical and biological characteristics, including the Baltic Proper, the Bothnian Sea, and the Bothnian Bay; the latter two together form the Gulf of Bothnia. In the Baltic Proper, extensive blooms of filamentous cyanobacteria (*Aphanizomenon* sp., *Dolichospermum* sp., and *Nodularia spumigena*) thrive under N-limited conditions during summer (25). These blooms play a major role in N cycling by fixing N_2_ and sustaining primary productivity. The Gulf of Bothnia is considered N replete and therefore not a beneficial region for N_2_ fixation. However, long-term monitoring reports an increase in the abundance and distribution of filamentous diazotrophic cyanobacteria (26), which is in agreement with declining N to P ratios in the Bothnian Sea in recent decades (27). Thus, the Gulf of Bothnia is a region of the Baltic Sea where N_2_ fixation could increase in importance.

Phytoplankton spring blooms, typically dominated by diatoms and dinoflagellates (28), and filamentous cyanobacterial summer blooms are sources for phytoplankton-derived particles. Such particles could provide a niche for NCDs, potentially prolonging N_2_ fixation and fueling primary production into the post-bloom phase. In the Baltic Sea, NCDs can contribute up to 43% of the relative abundance of *nifH* genes (encoding a subunit for the nitrogenase enzyme) (4, 7). Deltaproteobacteria (part of the phylum group, Thermodesulfobacteriota) are the dominant NCDs in the Baltic Sea, representing between 10% and 80% of all the NCDs in the particle-associated fraction (>3 µm), while the free-living (0.2–3 µm) community showed a dominance of Gammaproteobacteria (7). A recent study showed that NCDs are indeed active and accounted for up to 70% of *nifH* genes in the particle-associated fraction and that there was a difference in the NCD composition between the particle-associated and the free-living fraction (7), suggesting niche preference.

Here, we use *nifH* gene amplicon sequencing to assess diazotrophs associated with different particle fractions (suspended and sinking) sampled during two cruises across different primary productivity phases (productive and post-productive phases) in the Northern Baltic Proper, the Gulf of Bothnia, and the Gulf of Finland. Our results advance the understanding of particle-associated diazotrophs in the Baltic Sea by revealing how temporal dynamics, associated phytoplankton assemblages, and particle fraction structure the diazotrophic community.

## RESULTS

### Environmental data

**Fig 1:**
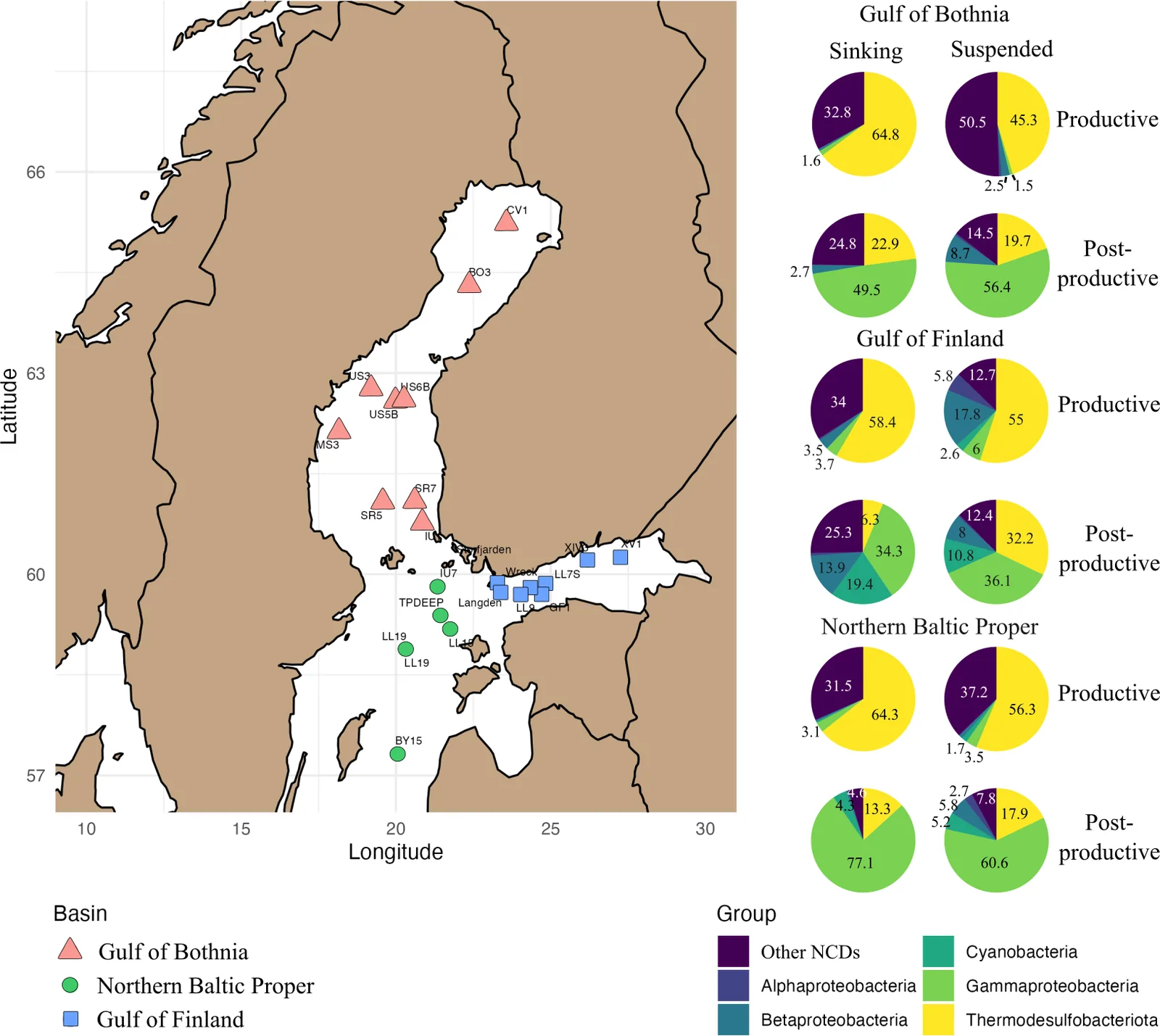
Map of sample locations in the Baltic Sea. Points are colored and shaped by basin. The circle diagrams show the relative abundance (in percentage) of major diazotroph groups, separated by particle fraction (sinking and suspended) and productivity phase (productive and post-productive). The map is made using RStudio—the packages: ggOceanMapsData, ggOceanMaps, maps.

Two cruises with R/V Aranda covered 22 sampling stations and two depths (5 and 30 m) in April and late May to early June in 2024. Four stations were in the Northern Baltic Proper, and one station in the Eastern Baltic Proper (BY15, IU7, LL15, LL19, TPDEEP) (latitude ~57°N to 59°N), now referred to solely as the Northern Baltic Proper. Eight stations (LL7S, GF1, Langden, LL9, Storfjarden, Wreck, XIV3, XV1) were in the Gulf of Finland (latitude ~61°N to 60°N), and eight stations were (BO3, CV1, IU1, MS3, SR5, SR7, US3, US5B) in the Gulf of Bothnia (latitude ~65°N to 61°N) (Fig. 1). The samples were separated into productivity phases based on the concentration of chlorophyll A (Chl A) and inorganic nutrients to Spilling et al. (29). Oxygen concentrations (0–40 m) ranged between 7.5 and 10 mL L^−1^ (335 to 450 µmol L^−1^) seawater, except for in the Gulf of Finland, where the concentration was ~5 mL L^−1^ (220 m-mmol L^−1^) (Table S1). In the Gulf of Bothnia in the productive phase, we observed a halocline at around 25 m, and in the post-productive phase at around 10 m (Table S1). On the contrary, we did not observe any halocline in the top 40 m of the Northern Baltic Proper or the Gulf of Finland. During the post-productive phase, higher temperatures (10°C–15°C) were measured in the surface waters (<5 m) of all basins with a thermocline between 15 and 20 m depth. In the productive phase, there was no apparent thermocline in any of the basins. ChlA, ammonium, nitrite, total nitrogen, phosphate, total phosphate, and silicate were similar across the basins and seasons (Table S1).

### Diazotroph composition: productive versus post-productive phase

**Fig 2:**
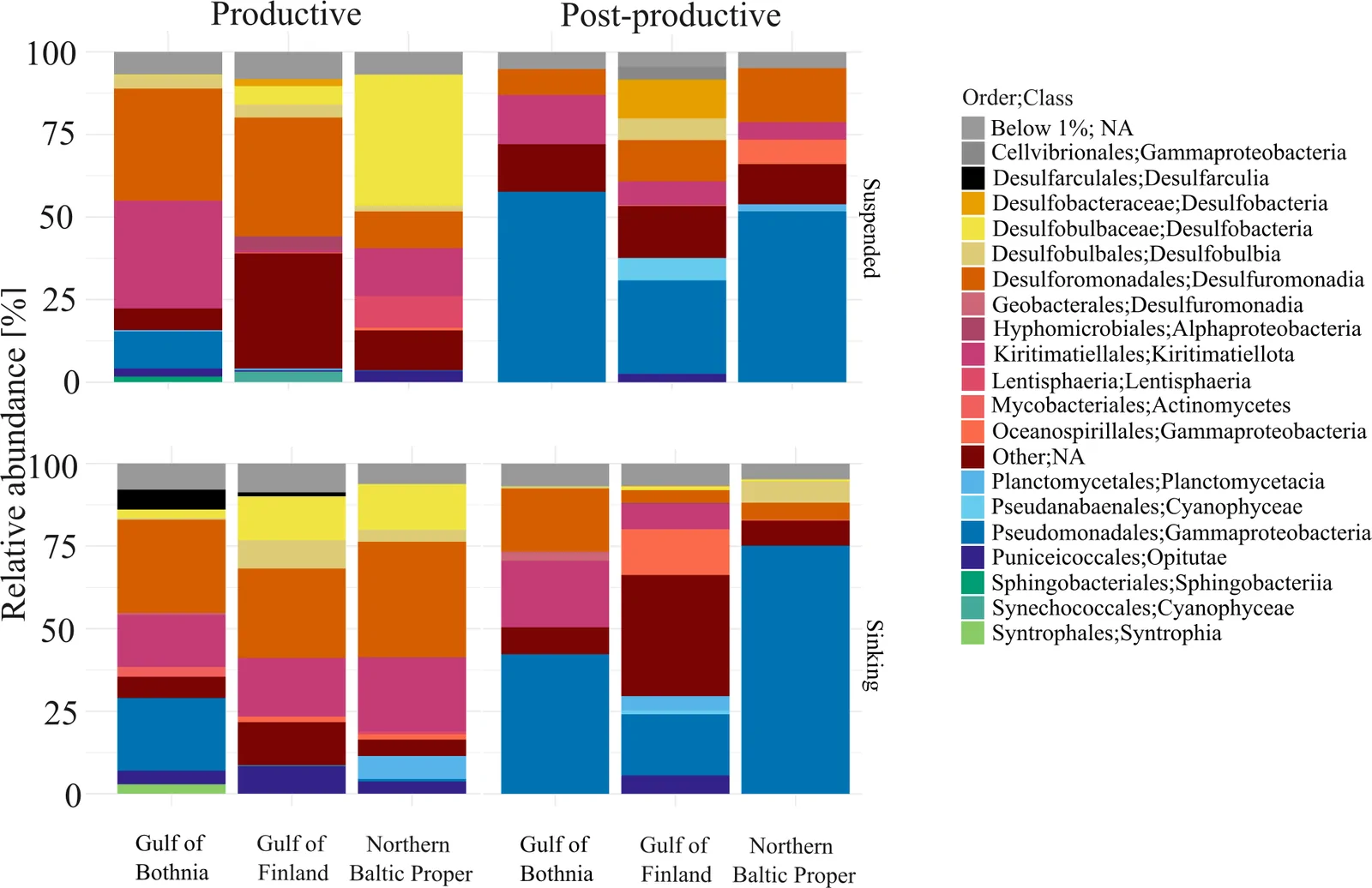
Relative abundance of the top 20 most abundant diazotroph (*nifH*) groups at the class and order levels, shown in percentage. The remaining taxa are grouped as “other; NA.” Data are separated into basin (Gulf of Bothnia, Gulf of Finland, and Northern Baltic Proper), productivity phase (productive and post-productive), and particle fraction (suspended and sinking).

In the *nifH* gene amplicon libraries, filamentous cyanobacterial sequences accounted for 0%–19%, whereas the remaining sequences, 81%–100% consisted of diverse NCDs (Fig. 1). These NCD sequences included Thermodesulfobacteriota (13%–65%), Gammaproteobacteria (<1%–77%), Betaproteobacteria (<1%–18%), Alphaproteobacteria (<1%–3%), and “other NCDs” (12%–50%), e.g., affiliated with Bacteroidota, Campylobacterota, Chlorobiota, and Planctomycetota. Between the productive and post-productive phases, all three basins showed similar diazotroph community shifts (Fig. 1), with low relative abundance of Gammaproteobacteria in the productive phase (<1%) and high relative abundance in the post-productive phase (up to 77%) (Fig. 2). Similar trends were observed for Cyanobacteria, which had low relative abundance (<1% in the productive phase) and higher relative abundance in the post-productive phase (up to 19%). Furthermore, the relative abundance of Betaproteobacteria was <1% in the productive phase and 14% in the post-productive phase. In contrast, the relative abundance of Thermodesulfobacteriota was higher in the productive phase (45%–65%) compared to the post-productive phase (6%–23%). “Other NCDs” also had a higher relative abundance in the productive phase (50%) compared to the post-productive phase (4%). Low-abundant amplicon sequence variants (ASVs) (<1%) accounted for 5%–15% of total relative abundances across samples (Fig. 2). Alpha-diversity (Shannon, Chao1, and Simpson) indices showed significant differences between the productive and post-productive phases (Table S2). Small variations were seen between depths (5 and 30 m) and stations within each phase, yet the overall trend was similar, with higher relative abundances of Thermodesulfobacteriota in the productive phase and higher relative abundances of Gammaproteobacteria in the post-productive phase (Fig. S1).

The high relative abundances of Gammaproteobacteria and Thermodesulfobacteriota are also reflected in the top 20 ASVs (Thermodesulfobacteriota 5%–10% in the productive phase; Gammaproteobacteria 5%–18% in the post-productive phase), contributing up to 20% of the total *nifH* gene amplicon libraries (Fig. S2). Among the 20 most abundant ASVs, 13 belonged to Thermodesulfobacteriota, spanning Desulfobacteria, Desulfuromonadia, and Desulfobulbia (Fig. S2). ASVs affiliated with Desulforomonadales and Desulfobulbaceae were dominant during the productive phase (10%–25% and 5%–40%, respectively). The relative abundance of Gammaproteobacterial ASVs (ASV1, ASV4, ASV7) was highest in the post-productive phase, with ASV1 contributing up to 55% (Fig. S2). Only one cyanobacterial ASV (ASV16, *Aphanizomenon* sp.) occurred among the top 20 ASVs.

**Fig 3:**
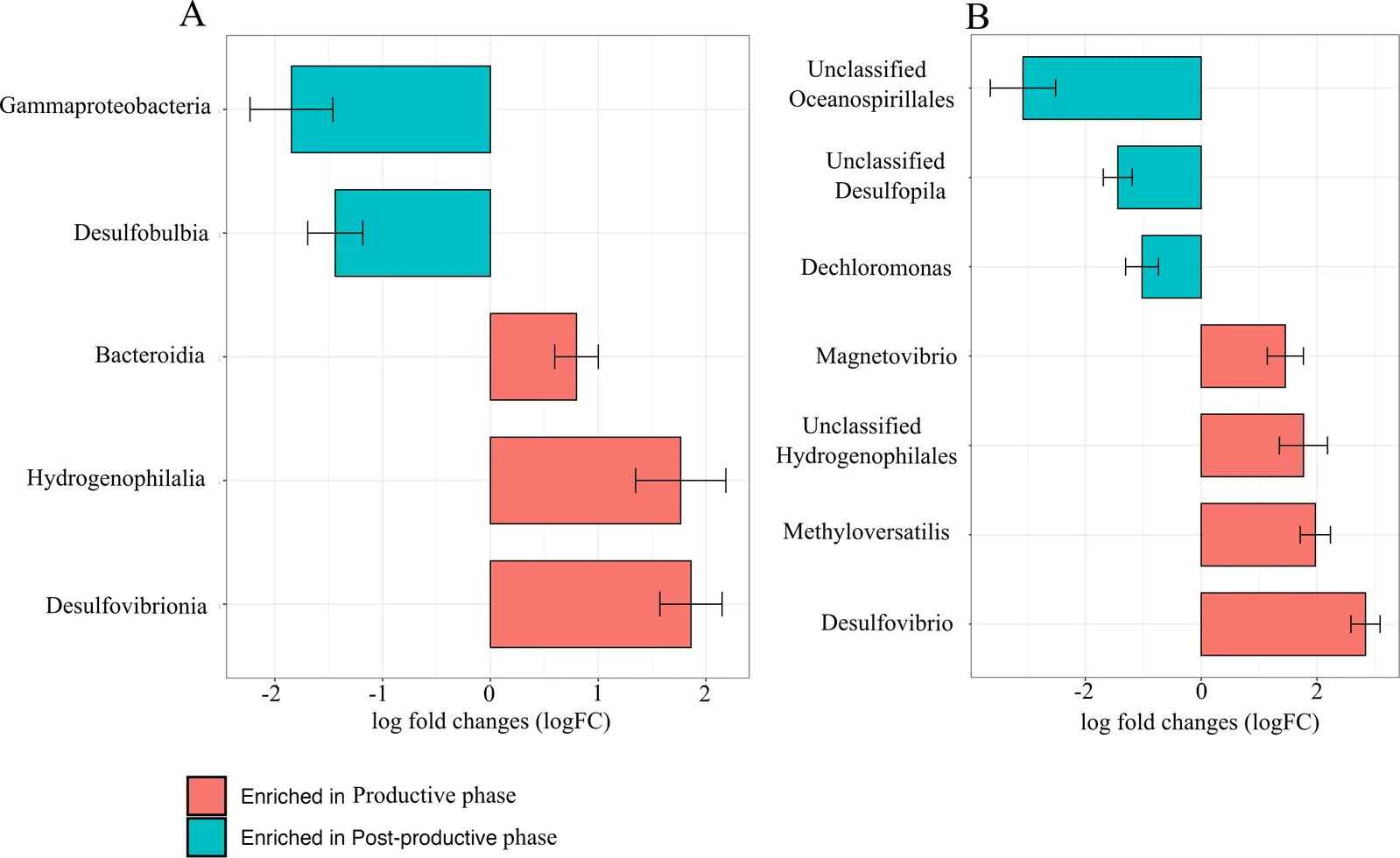
Differentially abundant taxa between the productive and post-productive phases on class (**A**) and order (**B**) levels. Log-fold changes (logFC) represent enrichment, with red being enriched in the productive phase and blue being enriched in the post-productive phase. Error bars represent standard error. Only taxa with statistically significant differences (adjusted *P* < 0.05) are shown.

Differential abundance analysis showed a significant (*P* < 0.05) enrichment of Bacteroidia, Hydrogenophilalia (unclassified Hydrogenophilales), Desulfovibrionia (Desulfovibrio), Methyloversatilis (Betaproteobacteria), and Magnetovibrio (Alphaproteobacteria) in the productive phase (Fig. 3A and B; Table S3). Conversely, Gammaproteobacteria (unclassified Oceanospirillales) and Desulfobulbia (unclassified Desulfopila) were significantly enriched (*P* < 0.05) in the post-productive phase (Fig. 3A and B; Table S3). On the ASV level, 13 ASVs showed differential abundance between the productive and post-productive phases, but only eight passed the sensitivity test (Table S3). Seven of the eight ASVs passing the sensitivity test were related to the phylum group Thermodesulfobacteriota. These included ASV52 and ASV462 (Desulfuromonadales and Desulfobulbaceae, respectively), which were enriched in the productive phase, and ASV105 (Desulfuromonadales), ASV102 (Desulfuromonadales), ASV7 (Desulfuromonadales), ASV67 (Wolinella), ASV4 (Desulfuromonadales), which were enriched in the post-productive phase. Finally, ASV1, a Gammaproteobacteria (Oceanospirillales), was enriched in the post-productive phase (Table S3).

### Diazotrophic composition: suspended versus sinking particles

**Fig 4:**
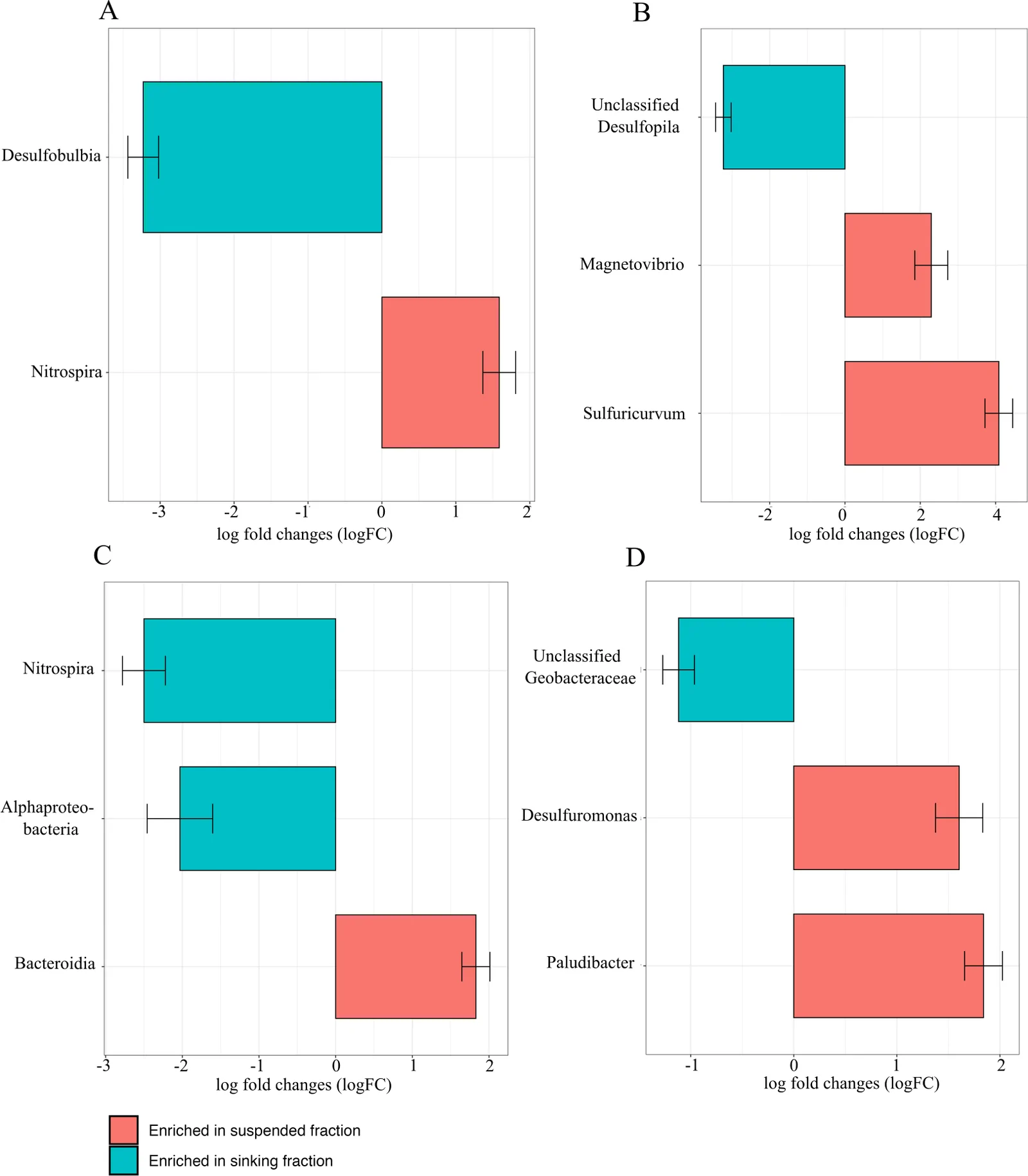
Differentially abundant taxa between the particle fractions (suspended and sinking), separated by productivity phase; productive phase (**A, B**) and post-productive phase (**C, D**). Log-fold changes (logFC) represent enrichment, with red being enriched in the productive phase, and blue being enriched in the post-productive phase. Error bars represent standard error. Only taxa with statistically significant differences (adjusted *P* < 0.05) are shown.

Suspended and sinking particles (sampled from marine snow catchers) contained the same major diazotrophic groups as described above, but their relative abundances varied (Fig. 2). Richness and diversity indices did not differ significantly between the particle fractions (Table S2). Differential abundance analysis of the particle fractions in the post-productive phase (Fig. 4C and D) showed a significant enrichment of Nitrospira and Alphaproteobacteria (*P* value < 0.05) in the sinking fraction and Bacteroidia in the suspended fraction (*P* value < 0.05) (Fig. 4C). On genus level, unclassified Geobacteraceae were significantly enriched in the sinking fraction, and Desulfuromonas and Paludibacter (Bacteroidia) in the suspended fraction (*P* value < 0.05) (Fig. 4D). On ASV level, only ASV17 (Desulfuromonadales) was significantly enriched in the suspended fraction (*P* value = 0.017) (Table S3). Differential abundance analysis of the particle fractions in the productive phase (Fig. 4A and B) revealed an enrichment of Desulfobulbia in the sinking fraction. The suspended fraction showed significant enrichment of Nitrospira, Alphaproteobacteria, Magnetovibrio, and Sulfuricurvum (Campylobacteria) (*P* value < 0.05) (Fig. 4A and B). On ASV level, only ASV12 was significantly enriched in the sinking fraction (ASV12, Desulfuromonadales) (*P* value < 0.005) (Table S3).

### Co-occurrence of phytoplankton and diazotrophs

**Fig 5:**
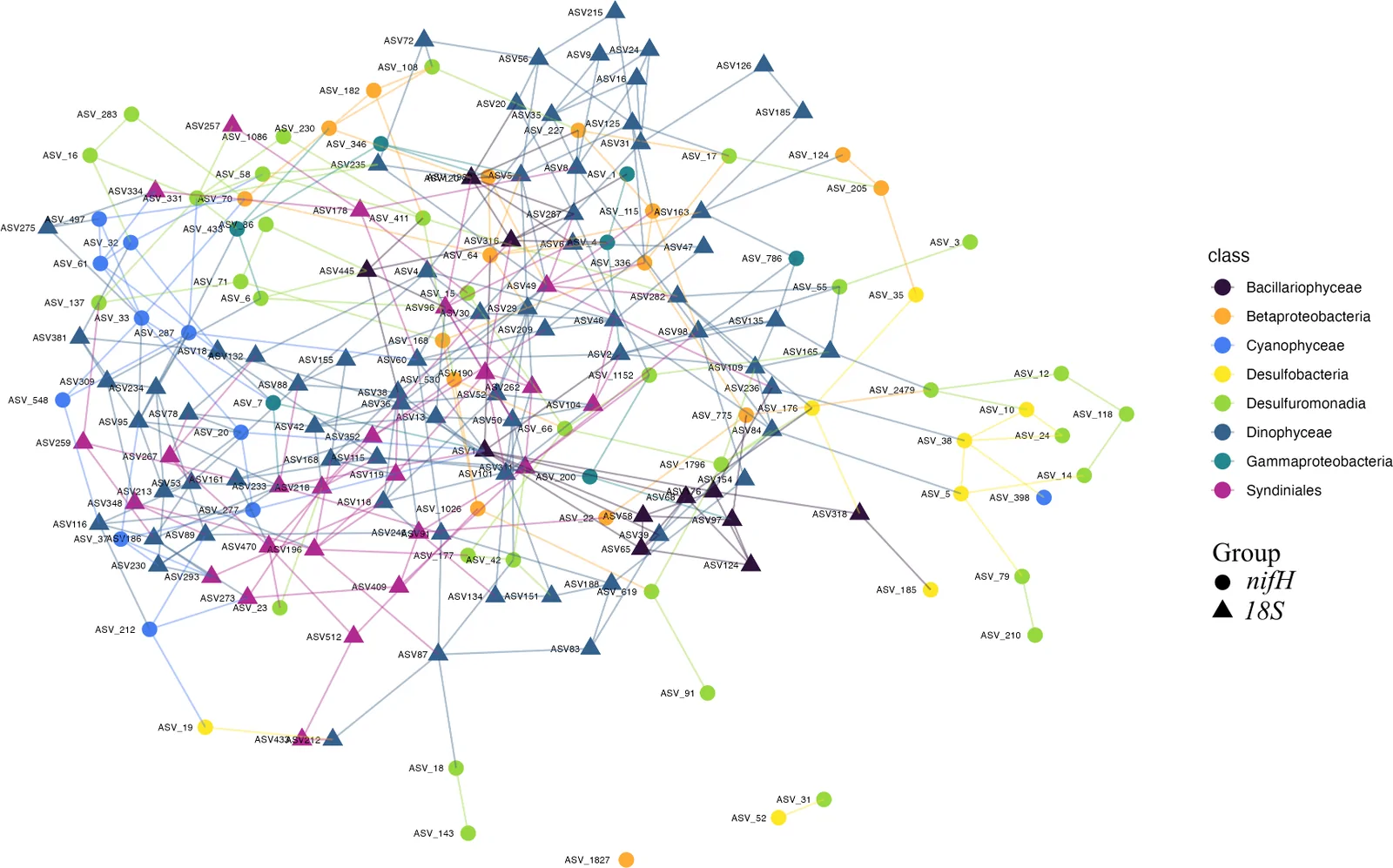
SpeicEasi network showing inferred associations among the top five *nifH* classes (Betaproteobacteria*,* Cyanophyceae*,* Desulfobacteria*,* Desulfuromonadia, and Gammaproteobacteria) and the top two 18S rRNA gene taxa (Diatoms [Bacillariophyceae] and Dinoflagellates [Dinophyceae and Syndiniales]) displaying significant correlations. Taxonomic groups are shown by color code. Edges represent an association between two ASVs using the Meinshausen-Buhlmann method. Shapes indicate whether the ASV is *nifH* or 18S.

Based on 18S rRNA gene amplicon sequencing, relative abundances of diatoms (Bacillariophyceae, Filosa-Thecofilosea, and Mediophyceae) and dinoflagellates (Dinophyceae and Syndiniales) varied between the productive and post-productive phases, across shallow (5 m) and deep samples (30 m), and particle fractions (Fig. S3). Diatoms had the highest relative abundance in the productive phase (up to 90%), and dinoflagellates in the post-productive phase (up to 90%). Co-occurrence network analysis revealed significant associations between *nifH* and 18S, indicating potential interactions between diazotrophs and eukaryotic plankton (Fig. 5). Diazotrophic taxa (Gammaproteobacteria, Betaproteobacteria, Desulfobacteria, Desulfuromonadia, and Cyanobacteria) were frequently associated with diatoms and dinoflagellates within several subnetworks (Fig. 5).

### Effects of productivity phase and basin on diazotrophic community structure

**Fig 6:**
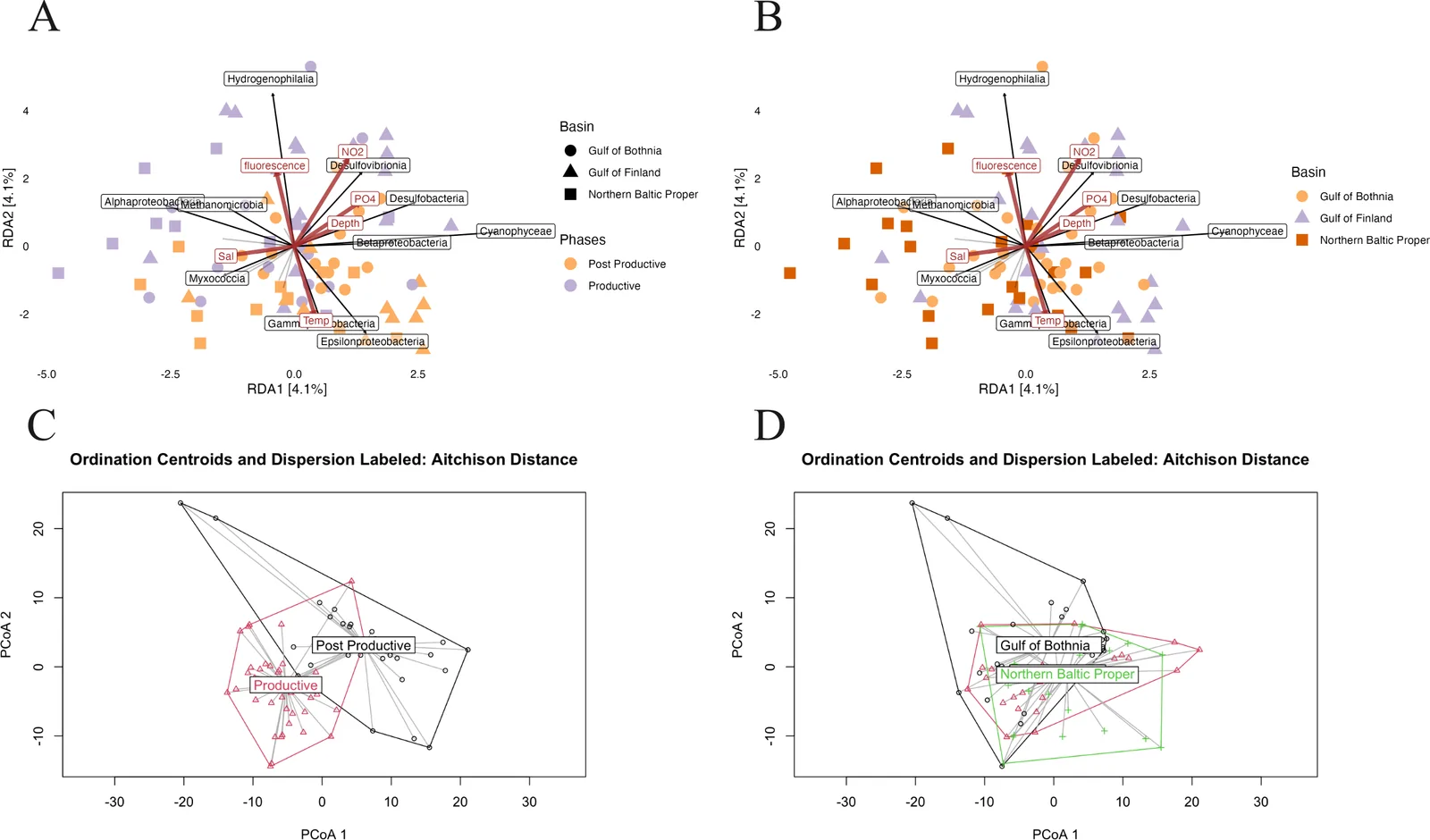
RDA showing the relationship between diazotrophic community composition and environmental variables across samples from the productive and post-productive phases (**A**) and basins (**B**). The red arrows indicate the direction and strength of environmental conditions (e.g., fluorescence, NO_2_, salinity, oxygen), and black text labels show the top 10 diazotrophic groups with the highest impact on the data set. PCoA illustrates differences in community dispersion between the compared groups (**C, D**). The productivity phase explained a significant portion of community variation according to adonis tests. Basin-specific ordinations indicated that the Gulf of Bothnia differs from the Gulf of Finland and Northern Baltic Proper, whereas the latter two largely overlap (note overlapping labels in the figure), consistent with non-significant adonis results.

Redundancy analysis (RDA) indicated that the productive and post-productive phases significantly influenced the diazotrophic community composition irrespective of basin (Fig. 6A). Salinity, NO_2_, and PO_4_ had an impact in separating the basins and diazotroph community (Fig. 6B). Adonis tests showed that the productivity phase explained a significant portion of the variation in the data set (*n* = 86, *P*.adj method = bonferroni, *R*^2^ = 0.031, *P* = 0.001), although interpretation should be made with caution due to a positive betadisper result, indicating unequal dispersion of variance between the two phases. Nonetheless, the clear separation of productive and post-productive samples in both RDA (Fig. 6A) and principal coordinates analysis (PCoA) centroids (Fig. 6C) supports that productivity phases exert a structuring influence on the diazotrophic community. In contrast, the effect of basin on diazotroph composition was weaker (Fig. 6B and D). Pairwise adonis tests revealed significant differences only between the Gulf of Bothnia and the Gulf of Finland (*n* = 62, *P*.adj method = bonferroni, *R*^2^ = 0.02, *P* = 0.002), and between the Gulf of Bothnia and the Northern Baltic Proper (*n* = 52, *P*.adj method = bonferroni, *R*^2^ = 0.02, *P* = 0.004), whereas no significant separation was detected between the Gulf of Finland and the Northern Baltic Proper (*n* = 54, *P*.adj method = bonferroni, *R*^2^ = 0.02, *P* = 0.134).

## DISCUSSION

### Productivity phase and phytoplankton composition structure the diazotroph community independent of basin

The clear separation between the productive and post-productive phases indicates that temporal variability is a dominating driver of diazotroph community structure, whereas basin-specific differences are comparatively small. This is directly or indirectly reflecting changes in the phytoplankton composition across productive and post-productive phases. Here, we observed a clear shift from diatom-dominated waters during the productive phase (yet, still varying presence of dinoflagellates, up to ~25%), while the post-productive phase was dominated by dinoflagellates. While productivity phases are statistically detectable factors in the data set, the variation remains unexplained. Yet, the spatial variation and interannual changes in phytoplankton composition are in line with previous studies in the Baltic Sea (30, 31). Notably, a composition shift was also seen in the diazotrophic community, where the productive phase was dominated by Thermodesulfobacteriota and the post-productive phase was dominated by Gammaproteobacteria, which suggests that the diazotroph community adapts to phytoplankton biomass and composition. Such interactions can occur on multiple levels. Firstly, there are tight links between phytoplankton and bacteria, as changes in phytoplankton composition alter the dissolved organic matter pool, mediating changes in the heterotrophic bacterial community composition (30). Secondly, the characteristics of particles are directly influenced by phytoplankton composition (10), influencing the environmental niches available for particle-associated NCDs. Thirdly, species-specific symbiosis between phytoplankton and NCDs may be significant. For example, a recent study has identified N_2_-fixing Gammaproteobacteria in symbiotic relationships with diatoms (32), and a similar symbiosis has been suggested in the Arctic (33). While we did find Gammaproteobacterial diazotrophs ASVs associated with diatom ASVs in our data set, it was evident that they also show significant co-occurrence with dinoflagellates. Thus, we do not see a clear sign of a significant diatom-Gammaproteobacteria symbiosis in the Baltic Sea and can only speculate whether this co-occurrence is important in the Baltic Sea. While exploratory, our results show a potential interaction between phytoplankton and NCDs; we also observed that the association is not fixed or limited to any specific symbiotic relationships. Nevertheless, these interactions will be critical to understand NCDs’ impact on N_2_ fixation in the Baltic Sea, and how this is integrated into the food web of the Baltic Sea.

Our study contributes to the increasing reports of high relative abundance of NCDs in *nifH* amplicon libraries from the Baltic Sea (3–7). Altogether, it remains an open question whether NCDs are actively fixing N_2_ and what their contribution could be to the total N budget; however, their consistent presence across stations and shifts with productivity phase suggests ecological relevance beyond genetic potential. In contrast, although filamentous diazotrophic cyanobacteria are considered the main diazotrophs in the Baltic Sea (1), their relative abundance in the *nifH* amplicon libraries in this study was low. This is likely due to the time of sampling, which was prior to the summer bloom, and the extended sampling into the Gulf of Bothnia, which has not traditionally been considered for N_2_ fixation.

In our data set, the most abundant cyanobacterial *nifH* sequences were Pseudanabaenales (*Pseudanabaena* spp.) and Synechococcales. *NifH* genes of the diazotrophic filamentous cyanobacteria *Pseudoanabaena* have been identified at high relative abundances in the Bothnian Sea (7). In general, Pseudanabaenales are not considered a major constituent of phytoplankton in the Baltic Sea (4, 34, 35), but there is contrasting evidence on their importance. In a study by Klawonn et al. (35), *Pseudoanabaena* was not detected as an active N_2_-fixer, but in another study during a late bloom, it showed high N_2_ fixation rates (34), indicating different seasonal or niche-specific traits. Furthermore, in this study, we did not find any sequences associated with UCYN-A, the nitroplast of a cosmopolitan unicellular alga, which has previously been detected in the western Baltic Sea (6, 7, 36).

The strong overlap between the basins in ordination space, together with high within-basin variance, suggests that spatial differences in diazotrophic composition are comparatively small relative to temporal (productivity phase-related) shifts. Temperature is known to be an important regulator of N_2_ fixation by filamentous cyanobacteria (37). In our study, Gammaproteobacteria were highly correlated with temperature, suggesting that temperature is an important factor also for structuring the NCD community. Given that many NCDs rely on carbon sources (9, 12, 38), their presence and activity may be linked to high organic matter content and stratified, occasionally hypoxic conditions typical of the Baltic Sea (39). Dedicated studies targeting NCDs will be relevant to investigate their ecological role and to clarify whether these groups contribute to active N₂ fixation.

### Enriched diazotrophs in suspended and sinking particles

Overall, the diazotroph community composition of the different particle fractions was similar, but with some significant taxon-specific differences. Particles collected during the post-productive phase had an overall higher relative abundance of Gammaproteobacteria in both the suspended and the sinking fractions. This has been reported before in studies from the Atlantic and Pacific Oceans, where Gammaproteobacteria were the dominating NCDs in both suspended and sinking fractions (16, 18, 19, 40). Despite the high presence of Gammaproteobacteria, differential abundance analysis did not indicate any enriched Gammaproteobacteria in either of the particle fractions. On the contrary, we saw fraction-specific enrichment of Nitrospira and Alphaproteobacteria in the sinking fraction, while Bacteroidia (Paludibacter) were enriched in the suspended fraction. Yet, among particles from the productive phase, ASVs belonging to Nitrospira and Alphaproteobacteria (Magnetovibrio) were enriched in the sinking fraction. This suggests that diazotrophs within the same class may adapt different metabolic strategies, depending on particle characteristics, or that at the ASV level, we see a more fine-scale adaptation. However, as this study is based on *nifH* amplicon analysis, further studies using nifH transcript data and/or *in situ* rate measurement are needed to validate whether there is a lifestyle adaptation.

Particles from the productive phase were dominated by groups belonging to Thermodesulfobacteriota (6%–65% relative abundance), which in general are highly present diazotrophs in the Baltic Sea (roughly 20%, up to 3.2 × 10^4^ transcripts L^−1^) (4, 7). They are typically anaerobic-sulfate-reducing microbes (9) and have been shown to be particle-associated on filters >3 µm and >10 µm in the Baltic Sea, but are also found in the free-living fraction (4, 7). The advantages of a particle-associated lifestyle may be influenced by the particle composition, porosity, and density. Characteristics of particles, such as the quality of carbon and the size of the anoxic core, are expected to be relevant for NCD activity on particles (8, 14). Modeling has determined that the size of a particle required for an anoxic core to develop is at least 400–500 µm (11, 14). However, previous studies have measured NCD N_2_ fixation on the surface of <300 µm in diameter particles (17, 18). In the Baltic Sea, particle sizes commonly reach >500 µm (on average) (41). Such larger particles often have a higher C:N ratio (42), which Desulfuromonadia (Phylum Thermodesulfobacteriota) seem to favor (16). In our study, we observed groups belonging to Thermodesulfobacteriota shifting from sinking particles during the productive phase to suspended particles during the post-productive phase on both the class and genus levels (Desulfuromonas, Desulfobulbia; *P* value < 0.05), and on the ASV level, where Desulfuromonadales was enriched in the sinking fraction (ASV_12) during the productive phase and the suspended fraction (ASV_17) during the post-productive phase. These findings underscore the role of particles as an ecological niche that supports NCDs, but also suggest metabolic versatility of NCDs, reflecting the variation of microhabitats. However, to resolve metabolic versatility, future studies could implement a combination of metagenomic/transcriptomic.

### Conclusion

Our study demonstrated that the diazotrophic community in the Baltic Sea is structured by productivity phases and phytoplankton community assemblages, rather than basin-scale variability. While suspended and sinking particle fractions harbored broadly similar communities, we still saw particle fraction-specific enrichments (on ASV level), underscoring that suspended and sinking particles provide distinct microhabitats. NCDs appear as potential players in N_2_ fixation across Baltic Sea basins, with Thermodesulfobacteria dominating during the productive phase and Gammaproteobacteria dominating in the post-productive phase, suggesting a temporal shift driven by environmental factors and biological interactions. Collectively, the findings emphasize that the Baltic Sea has a diverse assemblage of NCDs occupying specific environmental and particle-associated niches. Further understanding of the functional roles and activity of these groups will be critical for constraining N inputs in coastal systems under ongoing environmental change beyond the traditional focus on filamentous cyanobacteria.

## MATERIALS AND METHODS

### Sampling location

Samples were collected at 22 stations during two cruises with R/V Aranda from 14 to 25 April and 28 May to 8 June 2024, covering spring and early summer. The sampling area covered the Gulf of Bothnia, the Gulf of Finland, and the northern part of the Gotland Basin (latitude ~65°N to 57°N) (Fig. 1). Physical parameters (temperature, salinity, oxygen, fluorescence) were recorded at each station using a CTD (SBE911plus, fluorometer WET Labs ECO FLNTU). Samples for measuring inorganic nutrients (nitrite [NO_2_*^−^*], NO_2_*^−^* + nitrate [NO_3_*^−^*], phosphate [PO_4_^3−^], silicate [SiO_4_], total nitrogen [totN], and total phosphorus [totP]) were collected from 5 and 30 m depths using a carousel water sampler (SBE32) and analyzed using an automated continuous-flow nutrient analyzer (SAN++). Ammonia (NH_4_*^+^*) was measured manually with a spectrophotometer (GENESYS 10S UV-vis) during the first cruise and with a fluorometer coupled to the continuous-flow analyzer during the second cruise. Chl A was determined by filtration on a glass fiber filter (GF/F, Whatman), and the Chl A was extracted in 96% ethanol. Filters were stored in ethanol at −20°C until measurement on a spectrofluorometer (Cary Eclipse, Agilent Technologies) calibrated against known standards (Sigma-Aldrich). To identify the different phases of primary productivity (i.e., productive and post-productive phases) and classify samples accordingly, we used a combination of inorganic nutrients and Chl A concentration, as in Spilling et al. (29).

Water was collected from 5 and 30 m depth using a 30-L water sampler (Niskin bottle). The water was transferred, with a hose, to two custom-built settling tanks (referred to as marine snow catchers (MSC) in the text) (43, 44). The total volume of each MSC was 110 L, which was divided into two sections: where the top section (suspended fraction) was 104.5 L, and the bottom section (sinking fraction) was 5.5 L, enabling us to collect suspended and sinking particles as separate fractions (i.e., water being separated by particle sinking speed). Particles were allowed to settle for 2 h before 0.5 L of the sinking fraction and 1 L of the suspended fraction were collected into acid-washed polypropylene bottles. The samples were 0.2 µm-filtered (Sterivex capsule filter; Millipore, Germany, Darmstadt) using a peristaltic pump for downstream DNA extraction. After a maximum of 30 min filtration time, filters were stored at −80°C until extraction.

### Nucleic acid extraction and *nifH* amplicon sequencing

DNA was extracted with the FAST DNA SPIN Kit for Soil (MP Biomedicals). Lysis cocktail was prepared following the manufacturer’s instructions and injected into the Sterivex filter, with the addition of an incubation step with proteinase K (1% final concentration) at 55°C for 2 h. Amplification of *nifH* was done using a nested polymerase chain reaction (PCR) protocol (45, 46). The reverse primer nifH3 and the forward primer nifH4 were used for the first reaction (0.4 µM primers, 0.8 µg/µL BSA, and 10 mM MgCl_2_, annealing for 1 min at 54°C). The second reaction was done using the forward primer nifH1 and the reverse primer nifH2 with Illumina adapters (45, 46). The second reaction was done in triplicate. PCR products were checked with gel electrophoresis, and if a successful band was visible, the triplicate products were pooled and cleaned with Geneclean Turbo Kit (MP Biomedicals) and finally quantified using Qubit 2.0 fluorometer (Thermo Fisher Scientific Inc.). The PCR products were indexed with a subsequent PCR of 12 cycles using Nextera DNA Dual-index (N7-N5) and purified using the Agencourt AMPure XP PCR purification kit (Beckman Coulter). Samples were sequenced at Macrogen on an Illumina MiSeq v3.

Raw reads were processed in R, using the DADA2 pipeline v1.29 (47). Briefly, primers were cut using cutadapt (v2.6 [48]) with the following settings: one mismatch and no indels (disallows insertion and deletions entirely), and we discarded reads that did not have primers. Quality profiles were generated using FastQC (v0.11.9 [49]) and were followed by trimming. Finally, the reads were dereplicated and merged, and chimeras were removed. We taxonomically assigned sequences using the *nifH* DADA2-formatted database (50). In total, 3,601 ASVs with 5,533,843 total reads were obtained from 78 samples.

### 18S rRNA amplicon sequencing

PCRs for the amplification of 18S rRNA (eukaryotic community composition analysis) were performed using a two-step protocol using Phusion High-Fidelity PCR Master Mix (Thermo Scientific). 18S rRNA primers 454F (5′-CCAGCASCYGCGGTAATTCC-3′) and V4RB (5′-ACTTTCGTTCTTGATYRR-3′) were used (51) with overhang adaptors (0.5 µM). Cycling conditions were as follows: 1 cycle of 98°C for 30 s, 25 cycles of 98°C for 10 s, 53°C for 30 s, and 72°C for 15 s, followed by 1 cycle of 72°C for 2 min. Indexing and purification were performed as above. Samples were sequenced like *nifH*. Similar to *nifH* sequencing, 18S was processed in DADA2, and taxonomically annotated to the database PR^2^ (v5.0.0) (52).

### Statistical analysis

Richness and diversity indices (Shannon-Weaver, Simpson index of diversity, and Chao1 richness index) were calculated using ampvis2 v2.8 (53), and canonical correlation analysis was calculated using vegan v2.7-2 (54). Indices were calculated based on the whole data set, and differences between particle fractions, basins, and productivity phase (i.e., productive vs post-productive phase) were assessed using a Wilcoxon rank-sum test with adjusted *P* value (adjustment method Holm, confidence level 0.95). The R-package microViz v0.12.7 (55) was used for RDA, and the data were centered log-ratio normalized and grouped by productivity phase and basin. Beforehand, *envfit* was run to test for significant environmental factors influencing the community composition patterns. Collinearity of environmental variables was tested using *vif.cca*, and values >3 were removed for the final analysis. Lastly, the model was checked for significance using *anova.cca*, and adonis test (package pairwise adonis v.0.4.1 [56]) to analyze differences in our data set (i.e., between productivity phases, basin, and particle fraction).

Differential abundance analyses were done using the R-package *ancombc v2.6.0* (57), along with the wrapper *ancombc_pq* from the MiscMetabar package v0.12.1 (58). Briefly, the analysis was done using default settings, except for *prv_cut*, which was changed to 0.01 (i.e., retaining ASVs present in >1% of the samples). Holm’s correction was applied as the *P* value adjustment method, and only significant groups (*P* value < 0.05) passing the sensitivity test (i.e., adding pseudo-counts ranging from 0.01 to 0.5 to each taxon with zero counts) were shown. Linear regression models were performed, and sensitivity scores for each taxon were calculated. The analysis was run on (i) the whole data set to test differences between the productivity phases, and (ii) a split data set by particle fractions and productivity phase.

### Co-occurrence network

Co-occurrence networks were constructed to explore the connection between major diazotrophic and phytoplankton/protist groups. Sequence data were processed using the phyloseq package (v.1.54.0) (59). ASVs, from both *nifH* and 18S amplicon libraries, were merged into one phyloseq object, followed by removing ASVs less than 5% abundant in our data set. Finally, we filtered out nodes related to major diazotrophic (Gammaproteobacteria, Alphaproteobacteria, Betaproteobacteria, Cyanobacteria, Desulfobacteria, and Desulfuromonadia) and eukaryotic groups (Diatoms [Bacillariophyceae] and Dinoflagellates [Dinophyceae and Syndiniales]). SpiecEasi (v. 2.0.0) (60) was used to infer an ecological network to explore the *nifH*/18S ecological associations. We used the mb method, minimum lambda ratio 1e-2, nlambda = 20, and pulsar.params at 50. Networks were visualized in R using *igraph* (v. 2.2.1) (61) and *ggraph* (v. 2.2.2) (62).

## Supplementary Material

Reviewer comments

## Data Availability

nifH and 18S rRNA gene amplicon sequences are deposited at NCBI under BioProject number PRJNA1402399. All other data are available in the manuscript or as supplemental material.

## SUPPLEMENTAL MATERIAL

Supplemental figures and table spectrum.01386-26-s0001.docx Figures S1 to S3, and Tables S2 and S3.

Table S1 spectrum.01386-26-s0002.xlsx Environmental and biological factors.

OPEN PEER REVIEW reviewer-comments.pdf An accounting of the reviewer comments and feedback.

ASM does not own the copyrights to Supplemental Material that may be linked to, or accessed through, an article. The authors have granted ASM a non-exclusive, world-wide license to publish the Supplemental Material files. Please contact the corresponding author directly for reuse.
