# Supplementary material for: Composition of non-cyanobacterial diazotrophs in suspended and sinking particles is driven by productivity phase
Source: Microbiol Spectr. 2026 Aug 6;14(9):e01386-26. doi: 10.1128/spectrum.01386-26 (PMC13532352; doi:10.1128/spectrum.01386-26)
Supplement: Supplementary file 1 [file reviewer-comments.pdf]

AMERICAN SOCIETY FOR MICROBIOLOGY | Microbiology Spectrum

Composition of non-cyanobacterial diazotrophs in suspended and sinking particles is driven by productivity phase

Christian Reeder, Lisa Winberg von Friesen, Josephin Lemke, Kristian Spilling, and Hanna Farnelid

Corresponding Author(s): Christian Reeder, Linneuniversitetet

Review Timeline:

Submission Date:	April 24, 2026
Editorial Decision:	June 4, 2026
Revision Received:	June 18, 2026
Accepted:	June 23, 2026

Editor: Hongan Long

Reviewer(s): The reviewers have opted to remain anonymous.

Transaction Report:

(Note: With the exception of the correction of typographical or spelling errors that could be a source of ambiguity, letters and reports are not edited. The original formatting of letters and referee reports may not be reflected in this compilation.)

DOI: https://doi.org/10.1128/spectrum.01386-26

Re: Spectrum01386-26 (Composition of non-cyanobacterial diazotrophs in suspended and sinking particles is driven by productivity phase)

Dear Dr. Christian Furbo Reeder:

Thank you for the privilege of reviewing your work. Below you will find my comments, instructions from the Spectrum editorial office, and the reviewer comments.

Please return the manuscript within 60 days; if you cannot complete the modification within this time period, please contact me. If you do not wish to modify the manuscript and prefer to submit it to another journal, notify me immediately so that the manuscript may be formally withdrawn from consideration by Spectrum.

Revision Guidelines
To submit your modified manuscript, log into the submission site at https://spectrum.msubmit.net/cgi-bin/main.plex. Go to Author Tasks and click the appropriate manuscript title to begin. The information you entered when you first submitted the paper will be displayed; update this as necessary. Note the following requirements:

• Upload point-by-point responses to the issues raised by the reviewers in a file named "Response to Reviewers," NOT in your cover letter.
• Upload a compare copy of the manuscript (without figures) as a "Marked-Up Manuscript" file.
• Upload a clean .DOC/.DOCX version of the revised manuscript and remove the previous version.
• Each figure must be uploaded as a separate, editable, high-resolution file (TIFF or EPS preferred), and any multipanel figures must be assembled into one file.
• Any supplemental material intended for posting by ASM should be uploaded with their legends separate from the main manuscript. You can combine all supplemental material into one file (preferred) or split it into a maximum of 10 files with all associated legends included.

For complete guidelines on revision requirements, see our Submission and Review Process webpage. Submission of a paper that does not conform to guidelines may delay acceptance of your manuscript.

Data availability: ASM policy requires that data be available to the public upon online posting of the article, so please verify all links to sequence records, if present, and make sure that each number retrieves the full record of the data. If a new accession number is not linked or a link is broken, provide Spectrum production staff with the correct URL for the record. If the accession numbers for new data are not publicly accessible before the expected online posting of the article, publication may be delayed; please contact production staff (Spectrum@asmusa.org) immediately with the expected release date.

Publication Fees: For information on publication fees and which article types are subject to charges, visit our website. If your manuscript is accepted for publication and any fees apply, you will be contacted separately about payment during the production process; please follow the instructions in that e-mail. Arrangements for payment must be made before your article is published.

ASM Membership: Corresponding authors may join or renew ASM membership to obtain discounts on publication fees. Need to upgrade your membership level? Please contact Customer Service at Service@asmusa.org.

The ASM Journals program strives for constant improvement in our submission and publication process. Please tell us how we can improve your experience by taking this quick Author Survey.

Thank you for submitting your paper to Spectrum.

Sincerely,
Hongan Long
Editor
Microbiology Spectrum

Reviewer #1 (Comments for the Author):

The manuscript investigates the composition and temporal dynamics of non-cyanobacterial diazotrophs associated with suspended and sinking particle fractions across three Baltic Sea sub-basins. The topic is relevant, and the study provides useful descriptive information on particle-associated nifH assemblages in a brackish marine system. The spatial coverage across basins and the comparison between productive and post-productive phases are valuable.

However, several aspects of the interpretation and statistical analysis need to be strengthened or qualified.

Major comments

1. Functional interpretation exceeds the limits of DNA-based amplicon data.

The study is based on nifH gene amplicon sequencing and 18S rRNA gene amplicon sequencing. These data describe the relative composition of diazotrophic and eukaryotic assemblages, but they do not demonstrate nifH expression, nitrogenase activity, or N2 fixation rates. Nevertheless, several parts of the manuscript infer active metabolic strategies, ecological functions, and aggregate-associated nitrogen fixation from gene presence and relative sequence abundance.

The abstract and conclusion refer to "specific ecological strategies," "potential roles in aggregate-associated nitrogen fixation," and "different metabolic strategies." These statements should be toned down throughout the manuscript. The authors should consistently frame the findings as patterns in nifH gene assemblages or diazotrophic genetic potential, unless supported by nifH transcript data, metatranscriptomics, or direct 15N2 fixation measurements.

2. The PERMANOVA interpretation is weakened by unequal dispersion.

The manuscript reports that productivity phase significantly structures the diazotrophic community based on Adonis/PERMANOVA results. However, the authors also state that the betadisper result was positive, indicating unequal dispersion between the two phases. This is important because PERMANOVA can be sensitive to differences in within-group dispersion, and significant results may partly reflect dispersion effects rather than differences in group centroids.

The conclusion that productivity phase is the strongest or dominant structuring factor should therefore be presented more cautiously. The authors should either provide additional analyses that are robust to unequal dispersion, explicitly separate centroid effects from dispersion effects, or substantially qualify this interpretation.

3. Effect sizes are very small and should be interpreted cautiously.

The reported Adonis effect sizes are low, with R2 = 0.031 for productivity phase and R2 = 0.02 for basin comparisons. Although these effects may be statistically significant, they explain only a small fraction of the total community variation. Statements such as "strongest structuring factor" and "dominating driver" are therefore too strong unless placed in the context of the very limited explained variance.

The authors should revise the discussion to make clear that productivity phase is a statistically detectable factor in this dataset, but that most variation remains unexplained by the tested categorical variables.

4. Marine Snow Catcher fraction separation requires more methodological justification.

The manuscript states that particles were allowed to settle for two hours before collection of the suspended and sinking fractions. More detail is needed on the rationale for this settling time and on how efficiently the Marine Snow Catcher separated suspended from sinking material. Because the suspended fraction volume was much larger than the sinking fraction volume, carryover or disturbance during drainage could influence downstream comparisons between fractions.

The authors should clarify whether the separation efficiency was validated, whether slow-sinking aggregates or free-living cells could contribute to the sinking fraction, and how potential cross-contamination was minimized.

5. Co-occurrence networks should be interpreted as exploratory.

The co-occurrence network is based on correlations between compositional amplicon datasets. Although the authors used CLR transformation and FDR correction, correlation networks from amplicon data can still generate spurious associations due to compositionality, shared environmental drivers, and indirect correlations. The authors should avoid language implying biological interactions unless additional evidence is available. They should either justify the CLR-Spearman approach more explicitly or consider methods designed for compositional network inference, such as SPIEC-EASI or SparCC.

Minor comments

1. Line 118: The citation formatting "Spilling et al. (2019) (Spilling et al., 2019)" is redundant and should be corrected.

2. Lines 119-120: Oxygen concentrations are reported as mL L−1 seawater. Please consider also providing molar units, such as µmol L−1 or µmol kg−1, to facilitate comparison with other biogeochemical studies.

3. Please standardize the capitalization of Adonis/adonis throughout the manuscript.

4. Lines 267-270: The absence of UCYN-A sequences is interesting. The authors should clarify whether the nested nifH primer set used here has known biases or mismatches against UCYN-A or brackish UCYN-A-related lineages.

5. Please check consistency in the reported number of sampling stations. The Results mention 20 sampling stations, whereas the Methods mention 22 stations.

Overall, the dataset is potentially useful, but the manuscript should more clearly distinguish between descriptive nifH gene community patterns and evidence for active nitrogen fixation or metabolic function.

Reviewer #2 (Comments for the Author):

This manuscript presents an interesting and valuable investigation of non-cyanobacterial diazotrophs (NCDs) in the Baltic Sea, using nifH amplicon sequencing across different productivity phases and particle fractions. The finding that NCDs dominate the diazotrophic community is novel. The work is generally well structured, and the ecological interpretations are thoughtful. I have a few suggestions that could further strengthen the paper, mainly regarding quantification, PCR methodology, and figure formatting.

The study relies entirely on relative abundance data from nifH amplicon sequencing. This is particularly important when comparing productive vs. post-productive phases and different particle fractions, where total microbial biomass and activity likely differ. Please consider adding quantitative data (e.g., qPCR of nifH across samples) or, at minimum, discuss this limitation explicitly and temper the ecological interpretations accordingly.

The authors used a three-step PCR protocol for nifH amplification, which is less common than one-step or two-step approaches. I wonder whether this could introduce bias (e.g., chimera formation, differential amplification), especially when comparing different phases and particle types. The same protocol was applied to 18S rRNA, but that does not fully rule out marker-specific issues. Could the authors provide additional validation? A brief discussion of potential bias and why the three-step approach was chosen would be very helpful.

Figure 2 formatting：The upper right panel appears slightly misaligned with the left panels. Also, the font of the label "suspend" seems different from the rest (e.g., "sinking"). Please check and adjust these for consistency.

This manuscript presents an interesting and valuable investigation of non-cyanobacterial diazotrophs (NCDs) in the Baltic Sea, using *nifH* amplicon sequencing across different productivity phases and particle fractions. The finding that NCDs dominate the diazotrophic community is novel. The work is generally well structured, and the ecological interpretations are thoughtful. I have a few suggestions that could further strengthen the paper, mainly regarding quantification, PCR methodology, and figure formatting.

1. The study relies entirely on relative abundance data from *nifH* amplicon sequencing. This is particularly important when comparing productive vs. post-productive phases and different particle fractions, where total microbial biomass and activity likely differ. Please consider adding quantitative data (e.g., qPCR of *nifH* across samples) or, at minimum, discuss this limitation explicitly and temper the ecological interpretations accordingly.

2. The authors used a three-step PCR protocol for *nifH* amplification, which is less common than one-step or two-step (nested) approaches. I wonder whether this could introduce bias (e.g., chimera formation, differential amplification), especially when comparing different phases and particle types. The same protocol was applied to 18S rRNA, but that doesn't fully rule out marker-specific issues. Could the authors provide additional validation? A brief discussion of potential bias and why the three-step approach was chosen would be very helpful.

3. The upper-right panel of Figure 2 appears slightly misaligned with the left panels. Also, the font of the label "suspend" seems different from the rest (e.g., "sinking"). Please check and adjust these for consistency.

Reviewer #1 (Comments for the Author):

The manuscript investigates the composition and temporal dynamics of non-cyanobacterial diazotrophs associated with suspended and sinking particle fractions across three Baltic Sea sub-basins. The topic is relevant, and the study provides useful descriptive information on particle-associated nifH assemblages in a brackish marine system. The spatial coverage across basins and the comparison between productive and post-productive phases are valuable. However, several aspects of the interpretation and statistical analysis need to be strengthened or qualified.

We thank the reviewer for the positive feedback.

Major comments

1. Functional interpretation exceeds the limits of DNA-based amplicon data.

The study is based on nifH gene amplicon sequencing and 18S rRNA gene amplicon sequencing. These data describe the relative composition of diazotrophic and eukaryotic assemblages, but they do not demonstrate nifH expression, nitrogenase activity, or N2 fixation rates. Nevertheless, several parts of the manuscript infer active metabolic strategies, ecological functions, and aggregate-associated nitrogen fixation from gene presence and relative sequence abundance.

The abstract and conclusion refer to "specific ecological strategies," "potential roles in aggregate-associated nitrogen fixation," and "different metabolic strategies." These statements should be toned down throughout the manuscript. The authors should consistently frame the findings as patterns in nifH gene assemblages or diazotrophic genetic potential, unless supported by nifH transcript data, metatranscriptomics, or direct 15N2 fixation measurements.

We agree with the reviewer and have toned down statements in the abstract and the conclusion and have also checked similar statements throughout the manuscript.

See lines: 23, 27, 29, 309-311 and 331-332.

2. The PERMANOVA interpretation is weakened by unequal dispersion.

The manuscript reports that productivity phase significantly structures the diazotrophic community based on Adonis/PERMANOVA results. However, the authors also state that the betadisper result

was positive, indicating unequal dispersion between the two phases. This is important because PERMANOVA can be sensitive to differences in within-group dispersion, and significant results may partly reflect dispersion effects rather than differences in group centroids.

The conclusion that productivity phase is the strongest or dominant structuring factor should therefore be presented more cautiously. The authors should either provide additional analyses that are robust to unequal dispersion, explicitly separate centroid effects from dispersion effects, or substantially qualify this interpretation.

We thank the reviewer for highlighting this important limitation of PERMANOVA. We agree that the significant betadisper result indicates unequal within-group dispersion between productive and post-productive phases. However, we want to add that the ordination analyses (RDA and PCoA; Fig. 5) show consistent separation of productive and post-productive samples in multivariate space, suggesting that the observed pattern reflects both differences in centroid location and dispersion.

We have revised the manuscript to explicitly acknowledge this limitation we now describe productivity phase as a major descriptor of diazotrophic community variation rather than unequivocally the dominant structuring factor.

In addition, we have toned down a sentence in the abstract, where we stated productive phase as the strongest structing factor (line 23)

3. Effect sizes are very small and should be interpreted cautiously.

The reported Adonis effect sizes are low, with $R2 = 0.031$ for productivity phase and $R2 = 0.02$ for basin comparisons. Although these effects may be statistically significant, they explain only a small fraction of the total community variation. Statements such as "strongest structuring factor" and "dominating driver" are therefore too strong unless placed in the context of the very limited explained variance.

The authors should revise the discussion to make clear that productivity phase is a statistically detectable factor in this dataset, but that most variation remains unexplained by the tested categorical variables.

We agree that the effect size associated with productivity phase was relatively small ($R^2 = 0.031$), indicating that productivity phase explains only a modest proportion of the total variation in diazotrophic community composition.

We now revised the following sentence see line 238 – 239

4. Marine Snow Catcher fraction separation requires more methodological justification.

The manuscript states that particles were allowed to settle for two hours before collection of the suspended and sinking fractions. More detail is needed on the rationale for this settling time and on how efficiently the Marine Snow Catcher separated suspended from sinking material. Because the suspended fraction volume was much larger than the sinking fraction volume, carryover or disturbance during drainage could influence downstream comparisons between fractions.

The authors should clarify whether the separation efficiency was validated, whether slow-sinking aggregates or free-living cells could contribute to the sinking fraction, and how potential cross-contamination was minimized.

The method that was used in this study was reported by Riley et al. 2012 and the settling time is the default time determined by the original paper. The construction, validation and details of the marine snow catcher method have been described in this paper, as well as Lemke et al. [1], and we have now added the reference in the methods section (Line 370).

5. Co-occurrence networks should be interpreted as exploratory.

The co-occurrence network is based on correlations between compositional amplicon datasets. Although the authors used CLR transformation and FDR correction, correlation networks from amplicon data can still generate spurious associations due to compositionality, shared environmental drivers, and indirect correlations. The authors should avoid language implying biological interactions unless additional evidence is available. They should either justify the CLR-Spearman approach more explicitly or consider methods designed for compositional network inference, such as SPIEC-EASI or SparCC.

We agree with reviewer that using a method designed for compositional network inference is suitable. In the revised manuscript, we have redone the analysis using SPIEC-EASI. The methods section, results and discussion have been updated accordingly (Lines 434-444). In addition, we have added that the analyses should be interpreted as exploratory (Lines 266).

In connection with the new analysis, Figure 6 has been changed to the figure illustrated below, along with changed figure legend. Furthermore, Figure S4 (an additionally co-occurrence network) were removed, as it was redundant).

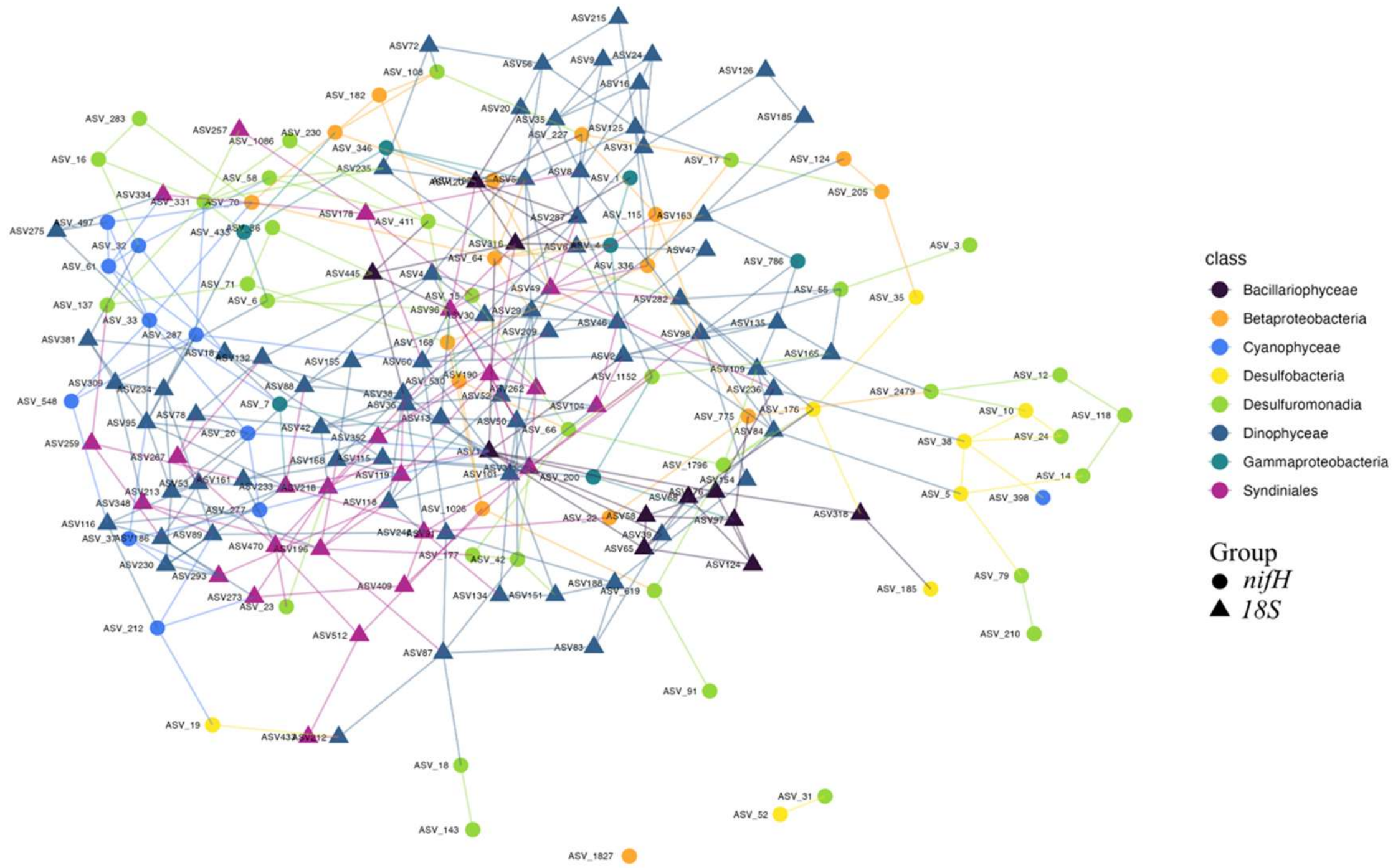

Figure 6. Speic-Easi network showing inferred associations among the top five *nifH* class (Betaproteobacteria*,* Cyanophycea*,* Desulfobacteria*,* Desulfuromonadia and Gammaproteobacteria*)* and top two 18S rRNA gene taxa (Diatoms (Bacillariophyceae) and Dinoflagellates (Dinophyceae and Syndiniales)) displaying significant correlations. Taxonomic groups are shown by color code. Edges represent an association between two ASVs using the Meinshausen-Buhlmann method. Shapes indicate whether the ASV is *nifH* or 18S.

Minor comments

1. Line 118: The citation formatting "Spilling et al. (2019) (Spilling et al., 2019)" is redundant and should be corrected.

Corrected (Lines 117).

2. Lines 119-120: Oxygen concentrations are reported as mL L−1 seawater. Please consider also providing molar units, such as µmol L−1 or µmol kg−1, to facilitate comparison with other biogeochemical studies.

Corrected. Oxygen ranged between 220 to 450 µmol L^{-1}

3. Please standardize the capitalization of Adonis/adonis throughout the manuscript.

Corrected

4. Lines 267-270: The absence of UCYN-A sequences is interesting. The authors should clarify whether the nested nifH primer set used here has known biases or mismatches against UCYN-A or brackish UCYN-A-related lineages.

The *nifH* primers are commonly used primers designed by [2,3], and are known to also detect UCYN-A. UCYN-A have been found in Arkona Basin (South Baltic Sea), using same primers prior [4], thus we do not expect a primer bias.

5. Please check consistency in the reported number of sampling stations. The Results mention 20 sampling stations, whereas the Methods mention 22 stations.

The number of sampling stations was 22 which has been corrected. Line 108.

Overall, the dataset is potentially useful, but the manuscript should more clearly distinguish between descriptive nifH gene community patterns and evidence for active nitrogen fixation or metabolic function.

We thank the reviewer for the valuable input.

Reviewer #2 (Comments for the Author):

This manuscript presents an interesting and valuable investigation of non-cyanobacterial diazotrophs (NCDs) in the Baltic Sea, using nifH amplicon sequencing across different productivity phases and particle fractions. The finding that NCDs dominate the diazotrophic community is novel. The work is generally well structured, and the ecological interpretations are thoughtful. I have a few suggestions that could further strengthen the paper, mainly regarding quantification, PCR methodology, and figure formatting.

We thank the reviewer for the positive feedback.

The study relies entirely on relative abundance data from nifH amplicon sequencing. This is particularly important when comparing productive vs. post-productive phases and different particle fractions, where total microbial biomass and activity likely differ. Please consider adding quantitative data (e.g., qPCR of nifH across samples) or, at minimum, discuss this limitation explicitly and temper the ecological interpretations accordingly.

We agree that quantitative approaches such as qPCR targeting the *nifH* gene of specific taxa could provide complementary information on diazotroph abundance but targeted approached could also miss the overall diazotroph diversity associated with different microniches. In line with the comment from the reviewer, we have revised the manuscript to explicitly acknowledge the limitation of our amplicon data and have been careful to interpret our results in terms of relative abundance and community structure rather than absolute abundance or activity. Please see line 310-311.

The authors used a three-step PCR protocol for nifH amplification, which is less common than one-step or two-step approaches. I wonder whether this could introduce bias (e.g., chimera formation, differential amplification), especially when comparing different phases and particle types. The same protocol was applied to 18S rRNA, but that does not fully rule out marker-specific issues. Could the authors provide additional validation? A brief discussion of potential bias and why the three-step approach was chosen would be very helpful.

Amplification by *nifH* was done using the standard nested-PCR protocol developed by Zehr and McReynolds 1989 and Zani et al. 2000 which is necessary for targeting low abundance gene targets. This protocol involves the use of two set of primers (outer and inner), *nifH3* and *nifH4* for first round of PCR, followed by *nifH1* and *nifH2* for the second round. To allow for next-generation high-throughput sequencing, an updated *nifH* protocol was developed where indexing with adaptors were added in a third step [5], which has since then been a standard protocol for *nifH* amplicon sequencing. Similarly, the 18S protocol is the same as what is typically used for amplicon studies [6]. Adding indexing primers is an essential step for multiplexing and can be associated with biases and artifacts, using an additional PCR-step with limited number of cycles, as we have done, is considered a way of limiting such biases.

Figure 2 formatting the upper right panel appears slightly misaligned with the left panels. Also, the font of the label "suspend" seems different from the rest (e.g., "sinking"). Please check and adjust these for consistency.

Corrected

References

1 Lemke, J. & Spilling, K. A low-cost method for collecting and measuring sinking speed of particles in coastal zones. *bioRxiv*, 2026.2006.2011.730824 (2026). https://doi.org/10.64898/2026.06.11.730824

2 Zehr, J. P. & McReynolds, L. A. Use of degenerate oligonucleotides for amplification of the nifH gene from the marine cyanobacterium Trichodesmium thiebautii. *Applied and environmental microbiology* **55**, 2522-2526 (1989).

3 Zani, S., Mellon, M. T., Collier, J. L. & Zehr, J. P. Expression of nifH genes in natural microbial assemblages in Lake George, New York, detected by reverse transcriptase PCR. *Applied and Environmental Microbiology* **66**, 3119-3124 (2000).
4 Reeder, C. F., Stoltenberg, I., Javidpour, J. & Löscher, C. R. Salinity as a key control on the diazotrophic community composition in the southern Baltic Sea. *Ocean Sci.* **18**, 401-417 (2022). https://doi.org/10.5194/os-18-401-2022
5 Farnelid, H. *et al.* Nitrogenase Gene Amplicons from Global Marine Surface Waters Are Dominated by Genes of Non-Cyanobacteria. *PLOS ONE* **6**, e19223 (2011). https://doi.org/10.1371/journal.pone.0019223
6 Sörenson, E., Farnelid, H., Lindehoff, E. & Legrand, C. Resource Partitioning Between Phytoplankton and Bacteria in the Coastal Baltic Sea. *Frontiers in Marine Science* **Volume 7 - 2020** (2020). https://doi.org/10.3389/fmars.2020.608244

Re: Spectrum01386-26R1 (Composition of non-cyanobacterial diazotrophs in suspended and sinking particles is driven by productivity phase)

Dear Dr. Christian Furbo Reeder:

Your manuscript has been accepted, and I am forwarding it to the ASM production staff for publication. Your paper will first be checked to make sure all elements meet the technical requirements. ASM staff will contact you if anything needs to be revised before copyediting and production can begin. Otherwise, you will be notified when your proofs are ready to be viewed.

Data Availability: ASM policy requires that data be available to the public upon online posting of the article, so please verify all links to sequence records, if present, and make sure that each number retrieves the full record of the data. If a new accession number is not linked or a link is broken, provide production staff with the correct URL for the record. If the accession numbers for new data are not publicly accessible before the expected online posting of the article, publication may be delayed; please contact ASM production staff immediately with the expected release date.

Publication Fees: For information on publication fees and which article types have charges, please visit ourwebsite. We have partnered with Copyright Clearance Center (CCC) to collect author charges. If fees apply to your paper, you will receive a message from no-reply@copyright.com with further instructions. For questions related to paying charges through RightsLink, please contact CCC at ASM_Support@copyright.com or toll free at +1-877-622-5543. CCC makes every attempt to respond to all emails within 24 hours.

ASM Membership: Corresponding authors may join or renew ASM membership to obtain discounts on publication fees. Need to upgrade your membership level? Please contact Customer Service at Service@asmusa.org.

Embargo Policy: A press release may be issued as soon as the manuscript is posted on theSpectrum Latest Articles webpage. The corresponding author will receive an email with the subject line "ASM Journals Author Services Notification" when the article is available online.

The ASM Journals program strives for constant improvement in our submission and publication process. Please tell us how we can improve your experience by taking this quick Author Survey.

Thank you for submitting your paper to Spectrum.

Sincerely,
Hongan Long
Editor
Microbiology Spectrum
