# Supplementary material for: Composition of non-cyanobacterial diazotrophs in suspended and sinking particles is driven by productivity phase
Source: Microbiol Spectr. 2026 Aug 6;14(9):e01386-26. doi: 10.1128/spectrum.01386-26 (PMC13532352; doi:10.1128/spectrum.01386-26)
Supplement: Supplementary file 2 [file spectrum.01386-26-s0001.docx]

Supplementary material

Supplementary figures

Figure S1. Overview of the diazotrophic community composition across stations, depths, particle fractions, and productivity phases. The top 20 ASVs are shown at class and order level, and the relative abundance is shown in percentage. Remaining taxa are grouped as “Other;NA”.

Figure S2. Top 20 ASVs, representing up to 20% of the total nifH read count. Data is separated into productivity phase (productive and post-productive) and particle fractions (suspended and sinking particles).

Figure S3. Relative abundance of dominant eukaryotic classes from 18S rRNA gene amplicon libraries, across productivity phases (productive and post-productive), depths (shallow: 5 m, deep: 30 m), and particle fractions. Stacked bar plots show the relative abundance of the top 10 eukaryotic classes (Bacillariophyceae (diatoms), Cryptophyceae, Dinophyceae (dinoflagellates), Filosa-Thecofilosea (diatoms), Litostomatea (ciliates), Mamiellophyceae, Mediophyceae (diatoms), Prymnesiophyceae, Spirotrichea (ciliates), and Syndiniales (dinoflagellates). The y-axis represents **relative abundance**, ranging from 0 to 1. The x-axis represents individual stations grouped into basins (GOF: Gulf of Finland, NBP: Northern Baltic Proper, GoB: Gulf of Bothnia).

**Supplementary Tables**

**Table S1**

See excel file

**Table S2** Pairwise comparisons of Shannon, Chao1, and Simpson diversity indices across basins, particle fractions, and productivity phases. Significance levels correspond to adjusted p-values (*p < 0.05, **p < 0.01, ***p < 0.001; ns = not significant). *#1 and #2 represent n-size in group1 and group2, respectively.

| **Richness between basins** | | | | | | |
| --- | --- | --- | --- | --- | --- | --- |
|  | **Group1** | **Group2** | **#1*** | **#2*** | **Adjusted p-value** | **significance** |
| Shannon | Gulf of Bothnia | Gulf of Finland | 28 | 31 | 0.015 | * |
| Shannon | Gulf of Bothnia | Northern Baltic Proper | 28 | 19 | 0.232 | ns |
| Shannon | Gulf of Finland | Northern Baltic Proper | 31 | 19 | 0.015 | * |
| Chao1 | Gulf of Bothnia | Gulf of Finland | 28 | 27 | 0.980 | ns |
| Chao1 | Gulf of Bothnia | Northern Baltic Proper | 28 | 23 | 0.136 | ns |
| Chao1 | Gulf of Finland | Northern Baltic Proper | 27 | 23 | 0.120 | ns |
| Simpson | Gulf of Bothnia | Gulf of Finland | 28 | 27 | 0.006 | ** |
| Simpson | Gulf of Bothnia | Northern Baltic Proper | 28 | 23 | 0.469 | ns |
| Simpson | Gulf of Finland | Northern Baltic Proper | 27 | 23 | 0.016 | * |
| **Richness between particle fractions** | | | | | | |
|  | **Group1** | **Group2** | **#1** | **#2** | **Adjusted p-value** | **Significance** |
| Shannon | Sinking | Suspended | 38 | 40 | 0.739 | ns |
| Chao1 | Sinking | Suspended | 38 | 40 | 0.678 | ns |
| Simpson | Sinking | Suspended | 38 | 40 | 0.732 | ns |
| **Richness over productivity phases** | | | | | | |
|  | **Group1** | **Group2** | **#1** | **#2** | **Adjusted p-value** | **Significance** |
| Shannon | Post-productive | Productive | 37 | 41 | 0.015 | * |
| Chao1 | Post-productive | Productive | 37 | 41 | 0.0004 | *** |
| Simpson | Post-productive | Productive | 37 | 41 | 0.034 | * |

**Tabel S3.** Differential abundance analysis. With respect to productive vs post-productive phases, positive log fold change (LFC)-values indicate enrichment in the productive phase, while a negative LFC indicates enrichment in the post-productive phase. With respect to particle fractions, a positive LFC indicates enrichment in the suspended fraction, while a negative LFC indicates enrichment in the sinking fraction. Note that the dataset was split into two when comparing particle fractions. Thus, particle fractions in the productive period were treated as one dataset, and particle fractions in the post-productive period were treated as a second dataset.

| **Productive vs. post-productive** | | | | | |
| --- | --- | --- | --- | --- | --- |
| **ASV** | **Order** | **Log fold change*** | **Standard deviation** | **q-value** | **Passed sensitivity test** |
| ASV_129 | Desulfuromonadales | 2.09 | 0.15 | 0.003 | FALSE |
| ASV_52 | Desulfuromonadales | 1.79 | 0.19 | <0.005 | TRUE |
| ASV_462 | Desulfobulbaceae | 1.04 | 0.15 | 0.013 | TRUE |
| ASV_62 | Desulfuromonadales | -1.51 | 0.25 | 0.009 | FALSE |
| ASV_399 | Pseudanabaena | -2.03 | 0.11 | 0.014 | FALSE |
| ASV_105 | Desulfuromonadales | -2.41 | 0.15 | <0.005 | TRUE |
| ASV_102 | Desulfuromonadales | -2.77 | 0.18 | <0.005 | TRUE |
| ASV_6 | Oceanospirillales | -2.95 | 0.32 | <0.005 | FALSE |
| ASV_7 | Desulfuromonadales | -3.33 | 0.33 | <0.005 | TRUE |
| ASV_67 | Wolinella | -3.66 | 0.11 | 0.007 | TRUE |
| ASV_4 | Desulfuromonadales | -4.02 | 0.44 | <0.005 | TRUE |
| ASV_1 | Oceanospirillales | -4.70 | 0.55 | <0.005 | TRUE |
| **Suspended vs. sinking fraction – productive phase** | | | | | |
| ASV_12 | Desulfuromonadales | -4.12 | 0.40 | <0.005 | TRUE |
| **Suspended vs. sinking fraction – post-productive phase** | | | | | |
| ASV_17 | Desulfuromonadales | 2.27 | 0.20 | 0.017 | TRUE |
